# Immuno-targeting the multifunctional CD38 using nanobody

**DOI:** 10.1038/srep27055

**Published:** 2016-06-02

**Authors:** Ting Li, Shali Qi, Mandy Unger, Yun Nan Hou, Qi Wen Deng, Jun Liu, Connie M. C. Lam, Xian Wang Wang, Du Xin, Peng Zhang, Friedrich Koch-Nolte, Quan Hao, Hongmin Zhang, Hon Cheung Lee, Yong Juan Zhao

**Affiliations:** 1 School of Chemical Biology and Biotechnology, Peking University Shenzhen Graduate School, Shenzhen 518055, China; 2 School of Biomedical Sciences, Li Ka Shing School of Medicine, The University of Hong Kong, Hong Kong, China; 3 Institute of Immunology, University Medical Center Hamburg-Eppendorf, Martinistr. 52, 20246 Hamburg, Germany; 4 Functional Laboratory, School of Medicine, Yangtze University, 1 Nanhuan Road, Jingzhou, Hubei 434023, China; 5 Department of Hematology, Shenzhen Second People’s Hospital, The First Affiliated Hospital of Shenzhen University, Shenzhen 518029, China; 6 Department of Oncology, Tongji Hospital, Tongji Medical College, Huazhong University of Science and Technology, Wuhan 430030, China; 7 Department of Biology, and Shenzhen Key Laboratory of Cell Microenvironment, South University of Science and Technology of China, Shenzhen 518055, China

## Abstract

CD38, as a cell surface antigen is highly expressed in several hematologic malignancies including multiple myeloma (MM) and has been proven to be a good target for immunotherapy of the disease. CD38 is also a signaling enzyme responsible for the metabolism of two novel calcium messenger molecules. To be able to target this multifunctional protein, we generated a series of nanobodies against CD38 with high affinities. Crystal structures of the complexes of CD38 with the nanobodies were solved, identifying three separate epitopes on the carboxyl domain. Chromobodies, engineered by tagging the nanobody with fluorescence proteins, provide fast, simple and versatile tools for quantifying CD38 expression. Results confirmed that CD38 was highly expressed in malignant MM cells compared with normal white blood cells. The immunotoxin constructed by splicing the nanobody with a bacterial toxin, PE38 shows highly selective cytotoxicity against patient-derived MM cells as well as the cell lines, with half maximal effective concentration reaching as low as 10^−11^ molar. The effectiveness of the immunotoxin can be further increased by stimulating CD38 expression using retinoid acid. These results set the stage for the development of clinical therapeutics as well as diagnostic screening for myeloma.

CD38 is a 46-kDa membrane glycoprotein with a short 20-aa N-terminal sequence, a single transmembrane segment and a 256-aa catalytic carboxyl domain. Decades of research documents that it is a novel multifunctional molecule, serving not only as a differentiation antigen on cell surface, but also, most surprisingly, as the dominant signaling enzyme responsible for the metabolism of two intracellular calcium messenger molecules, cyclic ADP-ribose (cADPR) and nicotinic acid adenine dinucleotide phosphate (NAADP) (reviewed in Lee^1^). It is well-established that the C-terminal domain possesses all the catalytic activities of the enzyme. Paradoxically, as a surface molecule, it has long been known that its catalytic C-terminal domain faces the extracellular space instead of the cytosol. How an ecto-enzyme can produce two second messenger molecules that function intracellularly is an unresolved conundrum that has been intensely investigated. A body of recent studies have now established the existence of intracellular CD38 (reviewed in Lee^2^) and even a Type III CD38 with its catalytic C-domain facing the cytosol^3^. The development of specific immuno-targeting tools for CD38, as described in this study, should facilitate the resolution of this topological paradox.

In addition to its intracellular signaling functions, CD38 is also a surface antigen, serving as a receptor for ligands such as CD4^4^,^5^ and CD31^6^,^7^. CD38 is ubiquitously expressed in many cells, especially in the immune cells, such as lymphocytes and monocytes (reviewed in Malavasi *et al*.^8^). The expression was found to be extremely high in some malignant cells, including multiple myeloma (MM), and chronic lymphoid leukemia. MM is a plasma-cell cancer characterized by accumulation of malignant cells in the bone marrow and production of a monoclonal immunoglobulin (M protein). It remains incurable, although the median overall survival rate has been increased to 5 years owing to the introduction of novel treatments^9^. Considering the huge differences of the expression between normal and myeloma cells, CD38 is thought to be a good drug target for cancer therapy^10^,^11^,^12^ and the human monoclonal antibody, daratumumab (Darzalex™) has recently been approved by the US FDA^13^.

Besides the success of daratumumab, several other monoclonal antibodies against CD38 are in clinical trials. Nanobodies, also called single domain antibodies, have attracted much attention for their therapeutic potential as a class of powerful next-generation antibodies^14^. They are much smaller and more stable than conventional full-length antibodies but retain equivalent antigen-binding capacity^15^.

In this study, we used the phage-display technology to generate a series of clones of nanobodies against CD38 and have developed highly efficient expression systems in bacteria to mass produce them. Using X-ray crystallography we identified their epitopes on CD38. To demonstrate their versatility, we engineered and produced specific chromobodies and an immunotoxin targeting MM cells with very high efficacy. The results should set the stage for developing clinical therapeutics as well as diagnostic screening for myeloma.

## Results

### Structural characterization of a panel of nanobodies recognizing different epitopes on the surface of CD38

The C-terminal domain of human CD38 (residue 45–300) was expressed and produced in a yeast expression system as previously described^16^. The purified recombinant protein was used as an antigen to immunize llamas. Phage display technology was used to obtain 19 positive clones against CD38. Seven of them were selected and produced in large scale for crystallography using an *E.coli* expression system as described in the Methods.

**Figure 1:**
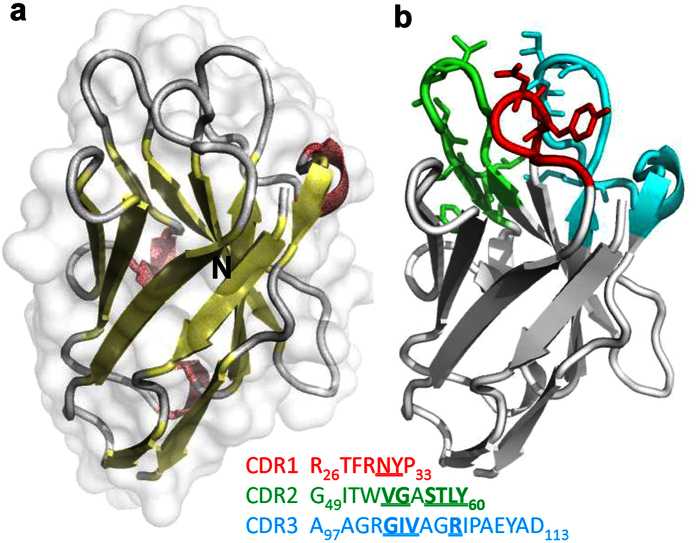
The overall structure of nanobody 1053. (**a**) A ribbon representation of the structure of 1053 with surface presented in a semi-transparent manner. (**b**) Ribbon structure of 1053 with three CDRs colored in different colors and residues forming closest contacts with CD38 are presented in the stick mode.

The secondary structure of one of these nanobodies is shown in Fig. 1. It contains prominent beta-sheets (yellow), making the molecule very compact and highly stable. Three short alpha helices (red), composing of three residues each, are also present. The three complementarity determining regions (CDRs) are located in three separate loops and are differently colored in Fig. 1b. The residues within these CDRs that form closest contacts of less than 3.9 Å with CD38 are rendered in sticks and listed in bold with underline.

**Figure 2:**
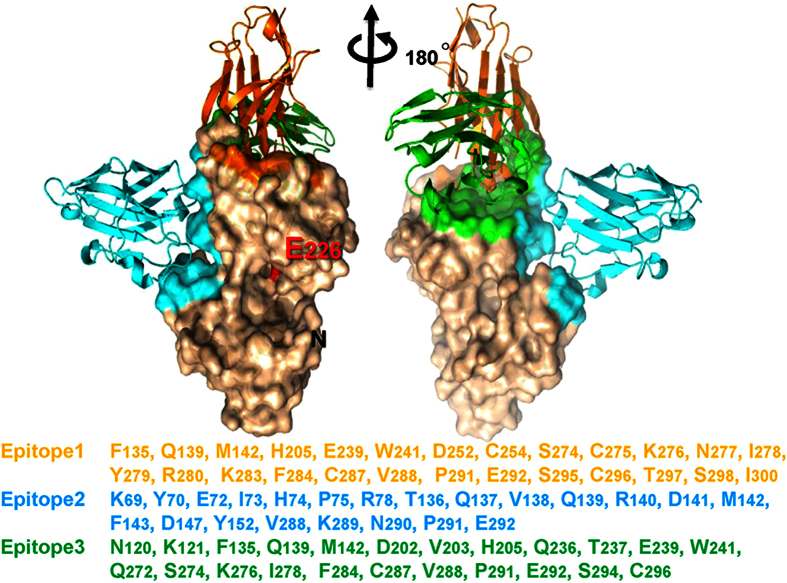
Two views of the crystal structure of CD38 complexed with nanobodies related by 180° rotation around a vertical axis. Three different nanobodies (shown in ribbon mode; orange: 375; cyan: 551; green: 1053) are composed with CD38 (shown in surface mode). The residues of the epitopes are colored both in the structure and in the listed sequence.

The crystal structures of seven different nanobodies complexed with CD38 were solved. Three of them are shown in Fig. 2 as a composite. Three separate epitopes are identified and are colored differently (orange, cyan or green). The residues composing the epitopes are also listed and colored correspondingly. Two of them (orange and green) are located close to C-terminal region of CD38. This region appears to be highly antigenic since it is also where the monoclonal antibody, HB-7, binds, as shown in the crystal structure we have previously solved^17^. Consistent with the long distance between the epitopes and the catalytic pocket^18^, none of these nanobodies shows any antagonistic effect on the enzymatic activity of CD38 (data are not shown).

An additional epitopic region (cyan) is distinct, which is located close to the middle of the molecule. The cleft at the middle of CD38 is where the active site pocket is located^18^, which can be seen in the left panel of Fig. 2, marked by the catalytic residue, E226 (colored red), present at the bottom of the pocket. The epitope is located on the opposite side of the pocket (right panel in Fig. 2).

### Chromobody 1053-FPs specifically stain CD38 on the cell surface

**Figure 3:**
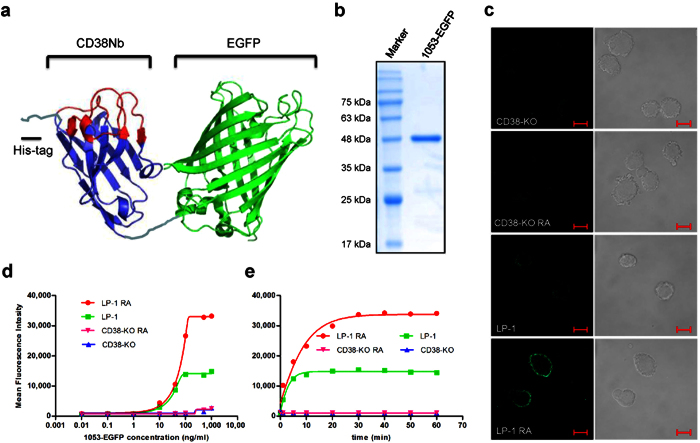
Designing and characterization of chromobody. (**a**) The structure of 1053-EGFP. (**b**) SDS-PAGE analysis of 1053-EGFP. (**c**) Confocal imaging of chromobody-stained cells. Bar: 10 μm. Dose curve (**d**) and time course (**e**) of live cell staining of 1053-EGFP analyzed by FACS. RA pre-treatment: 5 × 10^5^ cells/ml, 10 nM RA for 3 days; initial cell density for staining was 5 × 10^5^ cells/ml. The experiments were performed four times.

With the epitopes definitively identified, the panel of nanobodies developed in this study should be useful for immuno-targeting these regions for possible post translational modifications or for assessing interactions with regulatory factors. To facilitate quantification of binding of the nanobody to the surface expressed CD38, we engineered two fusion proteins by splicing EGFP or mCherry to the C-terminus of the nanobody, Nb1053, to create either a green (1053-EGFP) or a red (1053-mCherry) fluorescent nanobody. This type of fluorescent nanobody has been termed chromobody^19^. The construct of 1053-EGFP is diagrammed in Fig. 3a, the cDNA sequence of Nb1053, encoding 3 CDRs (red) and 4 framework residues (FRs, blue), was linked to EGFP (green), with a His_6_-tag in the N-terminus and a linker in between. This fusion gene was cloned into the prokaryotic expression vector, pET28a. The chromobody was expressed in *E.coli* BL21(DE3), mostly in the soluble fractions, and purified by sequential chromatography on Ni-NTA-agarose and Q-Sepharose ion exchange columns (see Material and Methods), yielding around 6 mg pure recombinant protein per liter of culture (Fig. 3b). By replacing EGFP with mCherry, a red colored chromobody was also prepared in a yeast system^16^, as described in Suppl. Fig. 1. The excellent yields of the preparations serve to illustrate the ease of mass scale production of these nanobodies as compared with monoclonal antibodies, which could be a deciding advantage for developing immuno-therapeutics.

To test the specificity and sensitivity of this chromobody, we used an MM cell line, LP-1, which expresses CD38 at high levels. As a negative control, we also constructed an LP-1 cell line with the CD38 gene knocked out (CD38-KO) using the CRISPR technique. The construction and validation of the CD38 knockout cell line is described in Suppl. Fig. 2. Figure 3c shows that the 1053-EGFP stained LP-1, but not CD38-KO, indicating the staining is specific for CD38. As retinoid acid (RA) was reported to be able to increase CD38 expression in HL-60 and other cells^20^,^21^, we tested the stimulation in LP-1. Indeed, CD38 expression was also stimulated by RA, not only in LP-1 but also in other two MM cell lines. The stimulation was in a concentration- and time-dependent manner (see Suppl. Fig. 3). After the RA treatment, the staining by 1053-EGFP was greatly enhanced in LP-1 but not CD38-KO (Fig. 3c), indicating again the staining by the chromobody was highly specific. Additionally, we have tested and found no cross-reactivity of 1053-EGFP to CD157, a CD38 paralogue protein (Suppl. Fig. 4).

Flow cytometry was used to quantify the staining. The concentration- and time- dependency of the increase in fluorescence intensity of the stained cells are shown in Fig. 3d. Significant fluorescence can be detected on the stained LP-1 cells with as low as 10 ng/ml of 1053-EGFP. At around 100–500 ng/ml of 1053-EGFP the fluorescence signals were saturated. The RA treatment increased the fluorescence of the cells by more than two folds, consistent with enhanced CD38 expression. Nonspecific binding was not observed on CD38-KO cells until the concentration went up to 1 μg/ml.

The staining of the cells with 1053-EGFP was done on ice to minimize possible internalization of the chromobody, even though its binding to the cells at room temperature was much faster (data not shown). On ice, the binding curves of LP-1 cells treated with or without RA plateaued at around 10–30 min (Fig. 3e). With these characterizations, all subsequent experiments were done using 500 ng/ml chromobody and 30 min incubation on ice. The ease of the procedure needs to be emphasized, as it requires just simple incubation of the cells with the chromobody, followed by analysis with flow cytometry directly. No fixing, washing or incubation with secondary antibody is required. As shown above, non-specific staining was virtually undetectable (cf. Fig. 3c).

**Figure 4:**
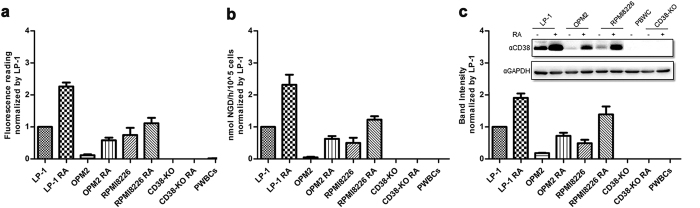
Different methods to quantify CD38 expression in different cell lines or normal PWBCs. (**a**) FACS following staining with 500 ng/ml 1053-EGFP at 4 °C for 30 min. (**b**) NGD assay of live cells. (**c**) Total CD38 in lysate was analyzed by Western blot, blotted with anti-CD38. Band intensity was quantified by ImageLab. All the data were normalized with the values in LP-1 cells. The experiments were performed five times.

Apart from simplicity, the sensitivity of the method was validated by comparison with two widely used techniques for measuring CD38; Western blot analysis and the NGD assay that is based on the enzymatic activity of CD38. The three assays differ in principle. The chromobody stains the cell surface CD38, while the Western blot analysis detects the total CD38 in cell lysates and the NGD assay measures the ADP-ribosyl cyclase activity of the surface CD38. As shown in Fig. 4, all three methods could detect CD38 signals in three different MM cell lines: LP-1, OPM2 and RPMI8226. All three methods are highly specific as none detected any signal from CD38-KO cells. Notably, the levels of CD38 in the cell lines detected by all three methods showed similar trend, with LP-1> RPMI8226> OPM2. Also, all three methods detected RA stimulation in all three cell lines, but not in the CD38-KO cells.

The chromobody method is highly sensitive as indicated by its readiness in detecting CD38 in the normal peripheral white blood cells (PWBCs), while the other two methods could not. Both monocytes and lymphocytes express much higher levels of CD38 than granulocytes (Suppl. Fig. 5), which is consistent with that reported previously^22^. Another advantage of chomobody-FACS method is that the amounts of cells required are the least of the three methods, which is an important factor when studying the limited samples from patients, as described below. Considering the cost and efficacy, we compared the chromobody with anti-CD38-FITC (T16, A07778 Beckman Coulter), which is routinely used in research and clinical tests. As shown in Suppl. Fig. 6, both the maximum signals and the linear range of the chromobody (right chart) are comparable to the commercial antibody (left chart). But the ease of preparation and the cost consideration (more than 300 times less), make the chromobody the method of choice.

### Immunotoxin 1053-PE38 efficiently kills MM cell lines in a CD38-dependent manner

The excellent sensitivity of the chromobody prompted us to consider developing a new therapeutic agent based on CD38 nanobody. We chose immunotoxin for two main reasons. First, immunotoxin is highly potent, even a single molecule of the toxin entering a cell can kill it^23^. Second, toxins suitable for this purpose, such as the bacterial PE38, have been well documented^24^.

**Figure 5:**
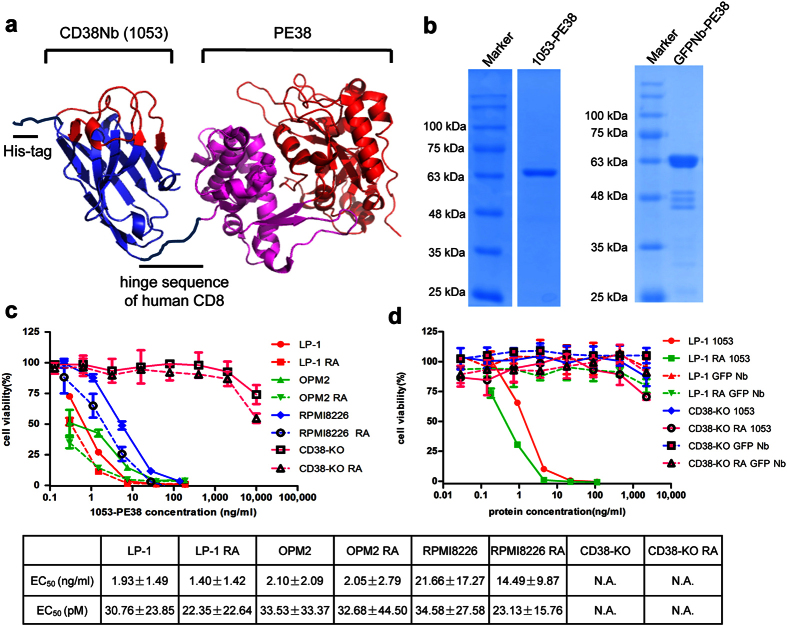
Designing and characterization of immunotoxin. (**a**) The structure of 1053-PE38. (**b**) SDS-PAGE analysis of pure 1053-PE38 and GFPNb-PE38. The cytotoxicity of 1053-PE38 (**c**) and GFPNb-PE38 (**d**), as a control, on MM cell lines, with or without RA pre-treatment measured by WST-1 assay and analyzed by Graphpad. The experiments were performed four times.

We employed the bacterial toxin domain, PE38 as the toxin part, which has been used in constructing various immunotoxins, including LMB-2, which is successful in a phase II clinic trial^25^. The construct of the immunotoxin is diagrammed in Fig. 5a, we used the hinge sequence of human CD8 to link the C-terminus of the nanobody (left, CDRs in red and FRs in blue) to the N-terminus of PE38 (right, translocation domain in magenta and enzymatic domain in red)^26^. A His_6_-tag from the backbone of pET28a was included to facilitate the purification process. The recombinant immunotoxin was expressed in *E.coli* BL21(DE3) and the majority was in soluble fraction. Around 4 mg of immunotoxin was purified from 1 L culture using Ni-NTA, anion exchange and size-exclusion chromatography (see Material and Methods) (Fig. 5b).

To test the cytotoxic activity of 1053-PE38, we employed a colorimetric assay based on cellular dehydrogenase-mediated reduction of the WST-1 tetrazolium salt to formazan. The results (Fig. 5c) show that all three MM cell lines are susceptible to killing by 1053-PE38 with EC_50_ values around 30 picomolar. The toxin was highly selective and did not affect the LP-1 cells not expressing CD38 (CD38-KO), unless at least 5 orders of magnitude higher concentration was used, indicating the cytotoxicity is strictly CD38-dependent. The potency of 1053-PE38 can be further increased by stimulating CD38 expression in the targeted cells. This was particularly notable in the RPMI8226 cells, showing a 68% reduction of the EC_50_ value, following treatment of the cells with RA (Table in Fig. 5).

To further test the non-specific toxicity of PE38-based immunotoxin, we prepared a control toxin composed with an unrelated nanobody against GFP^27^. The result shows that no significant cytotoxicity was observed in both CD38-positive or negative cells (Fig. 5d).

### Applications on the primary MM cells

**Figure 6:**
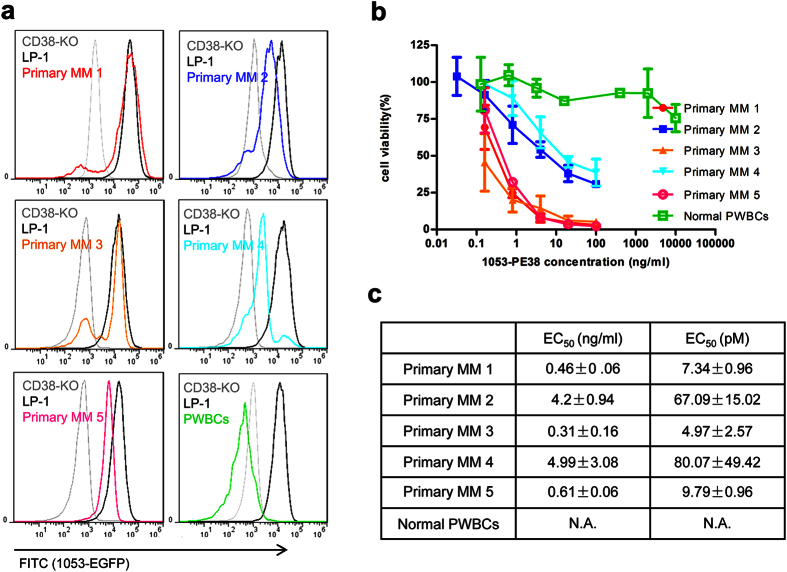
CD38 expression level of primary MM and the corresponding effects of the 1053-PE38. (**a**) CD38 expression in five different MM patient samples and PWBCs were analyzed by 1053-EGFP staining method, together with LP-1 and CD38-KO as relative expression controls. (**b**) The cytotoxicity of 1053-PE38 after 3-day treatment was analyzed by calcein staining followed by FACS. Three repeats were performed on each batch of samples.

To further test the efficacy and safety of the immunotoxin, we isolated MM cells from patient bone marrow samples and PWBCs from healthy donors as controls. We first quantified the amounts of cell surface CD38 by 1053-EGFP staining. The limited amounts of cells obtainable from patients made it suitable to use the chromobody quantification procedure developed in this study. As shown in Fig. 6a and Suppl. Fig. 7, both primary MM cells isolated from the patients and the LP-1 cell line have high levels of CD38 expression, while a very limited expression levels were detected on both lymphocytes and monocytes in the PWBCs isolated from healthy donors. Similar differences were also seen in the cytotoxicity of the immunotoxin. As shown in Fig. 6b,c and Suppl. Fig. 8, 1053-PE38 could kill the primary MM cells derived from the patients efficiently but had little effect on normal PWBCs. The EC_50_ values correlated to the expression levels of CD38. The expression levels of CD38 were higher in cells from patient-1, 3 and 5 than those from patient-2 and 4, and the corresponding EC_50_ values were around 7 pmol/L for the samples from patient-1, 3 and 5, lower than 70 pmol/L for those from patient-2 and 4.

## Discussion

CD38 is a novel protein serving multiple functions. Not only is it the dominant signaling enzyme in mammalian cells for mediating the mobilization of multiple intracellular calcium stores^2^, but it also functions as a surface antigen interacting with specific receptors, such as CD4^4^,^5^ and CD31^6^. Its expression level on activated T cells is an indicator of AIDS progression^28^ and has also been found to be highly expressed on myeloma cells^29^.

As a signaling enzyme, it is well established that one of its enzymatic products, cADPR, regulates the mobilization of the endoplasmic calcium stores, while another product, NAADP, targets the endo-lysosomal calcium stores instead^30^,^31^. Indeed, deletion of CD38 in mice has been shown to result in multiple defects, ranging from insulin secretion^32^, lymphocyte chemotaxis^33^ to depression of oxytocin release and social behavior^34^. How a single molecule can be involved in both extra- and intra-cellular functions has been a long unresolved conundrum. We believe that the development of specific and sensitive tool for targeting CD38 should be invaluable for resolving the conundrum, especially concerning its subcellular distribution and its membrane topology in relation with its multiple functions.

In this study, we have generated a series of nanobodies targeting three different epitopes on CD38, definitively identified by crystallography. We then engineered the nanobody to produce chromobody and showed that it is a highly sensitive tool for visualizing CD38 and quantifying its levels, with virtually no non-specificity. We have developed highly efficient expression system in *E.coli* that can be readily scaled up for mass producing the nanobody-based diagnostic agents as well as clinical therapeutics.

As an immuno-targeting tool for CD38, 1053-EGFP has the advantage of not only being highly sensitive, requiring limited amounts of cells, but also very simple to use (Figs 3 and 6). Both advantages have been illustrated in its ready detection of CD38 expression in primary cells isolated from individuals. Another important advantage of the chromobody is that it can be transfected and expressed inside cells, which should be useful for targeting intracellular CD38 that has been described in recent studies^19^. Indeed, chromobody has been used to visualize cytoskeletal dynamics in live cells^35^,^36^ and also can be used in single-molecule microscopy^37^.

The potential of using the nanobody to develope clinical therapeutics is also illustrated in this study. The results demonstrate that the engineered immunotoxin, 1053-PE38, selectively kills CD38-positive cells in a concentration-dependent fashion. The efficacy of this immunotoxin is in the picomolar range, with the particular EC_50_ values correlating with the amounts of surface CD38 expression in different cells types (Figs 4 and 5). The efficacy of 1053-PE38 compares favorably with the several monoclonal antibodies against CD38 for targeting MM cells that are currently in clinic trials, including daratumumab from GenMab/Johnson & Johnson^38^ and SAR650984 from Sanofi^39^. The EC_50_ values for daratumumab and SAR650984 are reported at around 1 nmol/L^38^ and 15–100 pmol/L^39^, respectively. Immunotoxins of CD38 have also been constructed based on the monoclonal antibodies, such as HB7^40^ and IB-4^41^, which shows excellent efficacy on MM cells and other CD38-overexpression cells. As discussed above, the nanobody-based immunotoxin does have several advantages over those based on monoclonal antibody, including simplicity for mass production. Also, as shown in this study, the compact molecular structure of the nanobodies stabilizes the molecules, making them suitable for long-term storage. The nanobody approach is thus a valuable alternative to monoclonal antibody.

Another notable feature of the nanobody is the remarkable specificity toward CD38. This is reflected not only in the chromobody staining, but also in the lack of cytotoxicity of 1053-PE38 towards cells expressing little or no CD38. As such, a wide safety window of around five order-of-magnitude difference in cytotoxic concentrations is seen between the MM cells and normal PWBCs (Figs 5 and 6), which should be a critical requirement for any therapeutic application.

Last but not least, that the nanobody is encoded by a single gene makes it amenable for improvement by protein engineering. Likewise, bivalent molecule targeting two different epitopes on CD38 can be constructed by introducing dimerizing motif such as FKBP^42^ and Fc domain^43^, which could increase its affinity for CD38 and prolongs its life time in serum. Nanobodies are also very suitable in making bispecific antibodies, which bind to two different antigens and convey great therapeutic potential^44^. As such, the nanobody approach is highly versatile for future development.

## Material and Methods

### Cell lines and samples from MM patients and healthy individuals

Myeloma cell lines, including LP-1, OPM2 and RPMI8226, which were kindly provided by Annie An (School of Pharmaceutical Sciences, Peking University) and verified by STR profiling test before experiments, were cultured in IMDM (for LP-1) or RPMI 1640 (OPM2 and RPMI8226) supplemented with 10% or 20% (OPM2) fetal calf serum (Life Technology), 1% penicillin–streptomycin solution (Life Technology). CD38 knockout LP-1 cell line was constructed by CRISPR technology according to previous report (Addgene, #48139)^45^. All the cells were maintained in a standard humidified tissue culture incubator with 5% CO_2_.

Primary malignant plasma cells were purified from MM patient bone marrow aspirates by negative selection using the RosetteSep human MM cell enrichment cocktail (StemCell Technologies) and Ficoll-Hypaque (Nycomed) density-gradient centrifugation according the manufacturers’ instructions. MM cells collected from the plasma/Ficoll interface were washed with PBS and applied to the tests of CD38 expression and cytotoxicity of immunotoxin.

Approximately 15 ml of peripheral blood was obtained from four healthy volunteers. Whole blood was applied to a Ficoll (Nycomed) gradient as previously described^3^. The isolated normal PBWCs were used as controls in the tests of CD38 expression and cytotoxicity of 1053-PE38.

The bone marrow samples were collected from MM patients. Informed consents were obtained from all patients concerning the use of the samples for research purposes. The sample collection and analysis *in vitro* were carried out in accordance with the recommendations in the research involving human biological materials of Shenzhen Second People’s Hospital. The protocol was approved by the Hospital Ethics Committee of Shenzhen Second People’s Hospital.

### Expression, purification, crystallization of the CD38-Nb complexes

Recombinant CD38 was prepared and purified as reported previously^16^,^46^. The purified protein was buffer-exchanged to 20 mM HEPES (pH 7.0), 50 mM NaCl, concentrated to 50 mg/ml and stored at −80 °C for later use. The nanobodies were cloned into pHEN2 vectors with an N-terminal pelB leading sequence and C-terminal His_6_- and C-myc tag. The proteins were expressed in *E.coli* BL21(DE3) or Rosetta(DE3) and purified by Ni-NTA affinity chromatography, followed by anion exchange Q or cation exchange SP. The proteins were buffer-exchanged to 20 mM HEPES (pH 7.0), 50 mM NaCl, concentrated to more than 20 mg/ml and stored at −80 °C for later use.

CD38 and nanobodies were mixed at equal molar ratio and diluted to 20 mg/ml. The protein mixtures were screened for crystallization conditions using hanging drop vapor diffusion method. Crystallization hints were further optimized for better crystals. The crystallization conditions for these protein complexes were listed in Suppl. Table 1. The crystals were harvested and soaked in cryo-protectant (c.f. Suppl. Table 1) and then flash frozen into liquid nitrogen. The diffraction data were collected at 100 K at BL17U at the Shanghai Synchrotron Radiation Facility and processed with HKL2000^47^. Molecular replacement was performed using the program Phaser^48^ from CCP4 suit^49^ and the wild type CD38 (PDB code 1YH3) was used as searching model. The model completion (especially the nanobody part) was performed using IPCAS as described^50^. Models were refined with Refmac^51^ and cycled with manual rebuilding in Coot^52^. TLS refinement^53^ was incorporated into the later stages of the refinement process for some of the complexes. Solvents were added automatically in Coot and then manually inspected and modified. The final models were analyzed with MolProbity^54^. Data collection and model refinement statistics were summarized in Table 1. The coordinates and structure factors were deposited in Protein Data bank with PDB code of 5F21, 5F1O and 5F1K for CD38-MU375, CD38-MU551 and CD38-MU1053, respectively.

### Construction, expression and purification of the immunotoxin, 1053-PE38 and GFPNb-PE38

Nb1053 was amplified from the phagemid pHEN2-1053 and subcloned to pET-28a(+) (Novagen) by *EcoR* I and *Hind* III, resulting an intermediate plasmid pET28a-1053. The sequence encoding hinge region of human CD8 and the truncated Pseudomonas exotoxin A (PE38) was amplified from the plasmid anti-CD11c IMMUNOTOXIN^26^ from Addgene (#22850) and subcloned into the above plasmid pET28a-1053 by *Hind* III and *Not* I. To construct GFPNb-PE38, Nb1053 was replaced with the gene encoding GFP nanobody, which was amplified from pOPINE-GFP nanobody^27^ (Addgene, #49172). The resulted plasmids were then transformed into *E.coli* BL21(DE3) to produce immunotoxins. The culture was harvested 12 h after induction by 0.5 mmol/L IPTG at 18 °C. More than 4 mg of immunotoxin could be purified from 1 L culture after purification using a standard Ni-NTA column (Qiagen), followed by a HiTrap Q column (GE Healthcare) and a Superdex 200 15/300 GL column (GE Healthcare) according to the manufacturers’ instructions.

### Construction, expression and purification of the chromobody, 1053-EGFP

To construct pET28a-1053-EGFP, the EGFP fragment, cut from pEGFP-N1 (Clontech) by *Hind* III and *Not* I, replaced PE38 in pET28a-1053. The expression conditions were similar to 1053-PE38. Around 6 mg of pure chromobodies were obtained from 1 L culture after purification using a standard Ni-NTA column (Qiagen), followed by a HiTrap Q HP 1 mL/5 mL (GE Healthcare) according to the manufacturers’ instructions.

### Measurement of ADP-ribosyl cyclase activity of CD38

The activity was measured by the colorimetric NGD assay^55^,^56^. Briefly, around 2 × 10^5^ live cells were applied to 50 μl of 100 μM NGD in PBS. Kinetic fluorescence reading (ex/em: 300/410 nm) was immediately started after adding NGD in an Infinite M200 PRO microplate reader (Tecan), maintained around 25 min and reCD38 was added to get a maximum reading (F_max_). The activity of the cell surface CD38 was calculated by the following equation: initial slope (F/h)  × 10 nmol NGD/(F_max_-F_min_), in which F_min_ is the initial fluorescence reading. The results were presented as nmol NGD/hour/10^5^ cells and normalized with the values of LP-1 cells.

### Measurement the amount of CD38 in cells

The expression levels of CD38 in different cells were measured by Western blot. Cells were or were not treated by 10 nM RA for 3 days and then lysed with the lysis buffer (PBS, 0.5% TRX-100, 2 mM EDTA, protease inhibitor cocktail (Roche)) and 40 μg total proteins were applied to SDS-PAGE. The blotting was performed using a homemade polyclonal antibody^3^ (αCD38), while anti-GAPDH (Sangon) as a loading control. Images were acquired by Chemidoc MP (Bio-rad) and analyzed by ImageLab (Bio-rad).

The amounts of CD38 on cell surface were measured by flow cytometry. 10^5^ cells were incubated with 500 ng/ml of 1053-EGFP for 30 min on ice and analyzed on CytoFLEX (Beckman Coulter). Data were analyzed by the software FlowJo.

### Measurement of cytotoxicity of the immunotoxin

To measure the cytotoxity of 1053-PE38 on different cells, WST-1 assay and FACS were used to evaluate the live cell numbers for cell lines and primary cells, respectively. Briefly, in WST-1 assay, 1 × 10^4^ cells were seeded together with different concentrations of immunotoxin in a 96-well plate. Seventy-two hours later, WST-1 reagent (Roche, 11644 807001) was added and incubated for 1 h at normal growth condition. The color intensity was read at 450 nm (reference wavelength: 690 nm) in an Infinite M200 PRO plate reader (Tecan). For primary MM cells and normal PWBCs, 1 × 10^5^ cells were seeded and incubated with immunotoxin as above for 72 h. Number of live cells was analyzed by calcein acetoxymethyl ester (calcein-AM) (C3100MP, Life Technology) staining and CytoFLEX (Beckman Coulter) according to the manufactures’ instructions. EC_50_ values were calculated using GraphPad Prism software and plotted.

## Additional Information

**How to cite this article**: Li, T. *et al*. Immuno-targeting the multifunctional CD38 using nanobody. *Sci. Rep.* **6**, 27055; doi: 10.1038/srep27055 (2016).

## Supplementary Material

Supplementary Information

## Figures and Tables

**Table 1:** Data collection and refinement statistics.

	**CD38-MU375**	**CD38-MU551**	**CD38-MU1053**
**Data collection**			
Space group	P2_1_2_1_2_1_	P2_1_2_1_2_1_	P2_1_2_1_2_1_
**Cell dimensions**			
*a, b, c* (Å)	45.149, 69.666, 123.476	33.141, 96.190, 143.254	88.552, 96.242, 133.777
α, β, γ(°)	90, 90, 90	90, 90, 90	90, 90, 90
Resolution (Å)	50~1.90 (1.97~1.90)	50~2.2 (2.28~2.2)	50~2.3 (2.38~2.30)
Unique reflections	29637(1958)	23341 (1897)	45209 (2716)
Completeness (%)	99.3(98.1)	99.7 (100.0)	99.7 (100)
Redundancy	6.1(6.1)	7.6(7.6)	9.8(9.1)
Wilson B-factor	20.75	26.66	28.87
*R*_merge_	0.069(0.614)	0.074(0.461)	0.092(0.576)
*I* /σ*I*	22.2(3.2)	22.6(5.0)	21.8(3.4)
**Refinement**			
Resolution (Å)	50~1.90	50~2.2	50~2.3
*R*_work_	0.1625	0.1679	0.198
*R*_*free*_	0.2108	0.2366	0.2313
No. atoms	3163	3165	6094
Protein	2874	2958	5771
ligands	16		
Water	273	207	323
Protein residues	362	369	727
**Ramachandran plot**			
Favored (%)	97.5	97.5	97.5
Outliers (%)	0.3	0.3	0.3
Average *B*-factors	27.74	34.92	42.58
Protein	26.8	34.55	43.07
ligands	27.98		
Water	37.59	40.08	33.8
**R.m.s. deviations**			
Bond lengths (Å)	0.009	0.009	0.01
Bond angles (°)	1.28	1.3	1.38

## References

[b1] Lee H. C. Cyclic ADP-ribose and nicotinic acid adenine dinucleotide phosphate (NAADP) as messengers for calcium mobilization. J Biol Chem 287, 31633–31640, doi: R112.349464/jbc.R112.349464 (2012). 22822066 10.1074/jbc.R112.349464 PMC3442497

[b2] Lee H. C. Cyclic ADP-ribose and NAADP: fraternal twin messengers for calcium signaling. Sci China Life Sci 54, 699–711, doi: 10.1007/s11427-011-4197-3 (2011). 21786193

[b3] Zhao Y. J., Lam C. M. & Lee H. C. The membrane-bound enzyme CD38 exists in two opposing orientations. Sci Signal 5, ra67, doi: 5/241/ra67 /scisignal.2002700 (2012). 22969159 10.1126/scisignal.2002700

[b4] Dianzani U. . Modulation of CD4 lateral interaction with lymphocyte surface molecules induced by HIV-1 gp120. Eur J Immunol 25, 1306–1311, doi: 10.1002/eji.1830250526 (1995). 7539755

[b5] Savarino A. . Human CD38 interferes with HIV-1 fusion through a sequence homologous to the V3 loop of the viral envelope glycoprotein gp120. FASEB journal : official publication of the Federation of American Societies for Experimental Biology 17, 461–463, doi: 10.1096/fj.02-0512fje (2003). 12551845

[b6] Deaglio S. . Human CD38 (ADP-ribosyl cyclase) is a counter-receptor of CD31, an Ig superfamily member. J Immunol 160, 395–402 (1998). 9551996

[b7] Deaglio S. . CD38/CD31 interactions activate genetic pathways leading to proliferation and migration in chronic lymphocytic leukemia cells. Molecular medicine 16, 87–91, doi: 10.2119/molmed.2009.00146 (2010). 19956559 PMC2785473

[b8] Malavasi F. . Evolution and function of the ADP ribosyl cyclase/CD38 gene family in physiology and pathology. Physiological reviews 88, 841–886, doi: 10.1152/physrev.00035.2007 (2008). 18626062

[b9] Hoy S. M. Carfilzomib Triple Combination Therapy: A Review in Relapsed Multiple Myeloma. Targeted oncology, doi: 10.1007/s11523-016-0428-7 (2016). 26972294

[b10] Stevenson G. T. CD38 as a therapeutic target. Molecular medicine 12, 345–346, doi: 10.2119/2006-00082.Stevenson (2006). 17380203 PMC1829201

[b11] Deaglio S., Aydin S., Vaisitti T., Bergui L. & Malavasi F. CD38 at the junction between prognostic marker and therapeutic target. Trends in molecular medicine 14, 210–218, doi: 10.1016/j.molmed.2008.02.005 (2008). 18403265

[b12] Vaisitti T. . Multiple metamorphoses of CD38 from prognostic marker to disease modifier to therapeutic target in chronic lymphocytic leukemia. Current topics in medicinal chemistry 13, 2955–2964 (2013). 24171772 10.2174/15680266113136660210

[b13] Magarotto V., Salvini M., Bonello F., Bringhen S. & Palumbo A. Strategy for the treatment of multiple myeloma utilizing monoclonal antibodies: A new era begins. Leukemia & lymphoma 57, 537–556, doi: 10.3109/10428194.2015.1102245 (2016). 26445358

[b14] Unciti-Broceta J. D., Del Castillo T., Soriano M., Magez S. & Garcia-Salcedo J. A. Novel therapy based on camelid nanobodies. Therapeutic delivery 4, 1321–1336, doi: 10.4155/tde.13.87 (2013). 24116915

[b15] Muyldermans S., Atarhouch T., Saldanha J., Barbosa J. A. & Hamers R. Sequence and structure of VH domain from naturally occurring camel heavy chain immunoglobulins lacking light chains. Protein engineering 7, 1129–1135 (1994). 7831284 10.1093/protein/7.9.1129

[b16] Munshi C. . Identification of the Enzymatic Active Site of CD38 by Site-directed Mutagenesis. Journal of Biological Chemistry 275, 21566–21571, doi: 10.1074/jbc.M909365199 (2000). 10781610

[b17] Zhao Y. J., Zhang H. M., Lam C. M., Hao Q. & Lee H. C. Cytosolic CD38 protein forms intact disulfides and is active in elevating intracellular cyclic ADP-ribose. J Biol Chem 286, 22170–22177, doi: M111.228379/jbc.M111.228379 (2011). 21524995 10.1074/jbc.M111.228379 PMC3121361

[b18] Liu Q. . Crystal Structure of Human CD38 Extracellular Domain. Structure 13, 1331–1339, doi: 10.1016/j.str.2005.05.012 (2005). 16154090

[b19] Schornack S. . Protein mislocalization in plant cells using a GFP-binding chromobody. The Plant journal : for cell and molecular biology 60, 744–754, doi: 10.1111/j.1365-313X.2009.03982.x (2009). 19686537

[b20] Mehta K. . Involvement of retinoic acid receptor-alpha-mediated signaling pathway in induction of CD38 cell-surface antigen. Blood 89, 3607–3614 (1997). 9160665

[b21] Prus E. & Fibach E. Retinoic acid induction of CD38 antigen expression on normal and leukemic human myeloid cells: relationship with cell differentiation. Leukemia & lymphoma 44, 691–698, doi: 10.1080/1042819031000060564 (2003). 12769347

[b22] Coetzee L. M., Tay S. S., Lawrie D., Janossy G. & Glencross D. K. From research tool to routine test: CD38 monitoring in HIV patients. Cytometry. Part B, Clinical cytometry 76, 375–384, doi: 10.1002/cyto.b.20478 (2009). 19422053

[b23] Kreitman R. J. Immunotoxins for targeted cancer therapy. The AAPS Journal 8, E532–E551, doi: 10.1208/aapsj080363 (2006). 17025272 PMC2761061

[b24] Jiang H. . Purification of clinical-grade disulfide stabilized antibody fragment variable—Pseudomonas exotoxin conjugate (dsFv-PE38) expressed in Escherichia coli. Appl Microbiol Biotechnol 97, 621–632, doi: 10.1007/s00253-012-4319-2 (2013). 22890777

[b25] Kreitman R. J. . Complete remissions of adult T-cell leukemia with anti-CD25 recombinant immunotoxin LMB-2 and chemotherapy to block immunogenicity. Clinical Cancer Research, doi: 10.1158/1078-0432.ccr-15-1412 (2015). PMC6314476 26350263

[b26] Huarte E. . Depletion of dendritic cells delays ovarian cancer progression by boosting antitumor immunity. Cancer research 68, 7684–7691, doi: 10.1158/0008-5472.CAN-08-1167 (2008). 18768667 PMC2742361

[b27] Kubala M. H., Kovtun O., Alexandrov K. & Collins B. M. Structural and thermodynamic analysis of the GFP:GFP-nanobody complex. Protein science : a publication of the Protein Society 19, 2389–2401, doi: 10.1002/pro.519 (2010). 20945358 PMC3009406

[b28] Liu Z. . Elevated CD38 antigen expression on CD8+ T cells is a stronger marker for the risk of chronic HIV disease progression to AIDS and death in the Multicenter AIDS Cohort Study than CD4+ cell count, soluble immune activation markers, or combinations of HLA-DR and CD38 expression. Journal of acquired immune deficiency syndromes and human retrovirology : official publication of the International Retrovirology Association 16, 83–92 (1997). 10.1097/00042560-199710010-00003 9358102

[b29] Leo R. . Multiparameter analyses of normal and malignant human plasma cells: CD38++, CD56+, CD54+, cIg+ is the common phenotype of myeloma cells. Annals of hematology 64, 132–139 (1992). 1373957 10.1007/BF01697400

[b30] Lee H. C. & Aarhus R. Functional visualization of the separate but interacting calcium stores sensitive to NAADP and cyclic ADP-ribose. J Cell Sci 113 Pt 24, 4413–4420 (2000). 11082034 10.1242/jcs.113.24.4413

[b31] Calcraft P. J. . NAADP mobilizes calcium from acidic organelles through two-pore channels. Nature 459, 596–600, doi: 10.1038/nature08030 (2009). 19387438 PMC2761823

[b32] Kato I. . CD38 disruption impairs glucose-induced increases in cyclic ADP-ribose, [Ca2+]i, and insulin secretion. J Biol Chem 274, 1869–1872 (1999). 9890936 10.1074/jbc.274.4.1869

[b33] Partida-Sanchez S. . Regulation of dendritic cell trafficking by the ADP-ribosyl cyclase CD38: impact on the development of humoral immunity. Immunity 20, 279–291 (2004). 15030772 10.1016/s1074-7613(04)00048-2

[b34] Jin D. . CD38 is critical for social behaviour by regulating oxytocin secretion. Nature 446, 41–45, doi: 10.1038/nature05526 (2007). 17287729

[b35] Van Audenhove I. . Mapping cytoskeletal protein function in cells by means of nanobodies. Cytoskeleton 70, 604–622, doi: 10.1002/cm.21122 (2013). 23818458

[b36] Rocchetti A., Hawes C. & Kriechbaumer V. Fluorescent labelling of the actin cytoskeleton in plants using a cameloid antibody. Plant methods 10, 12, doi: 10.1186/1746-4811-10-12 (2014). 24872838 PMC4036722

[b37] Platonova E. . Single-molecule microscopy of molecules tagged with GFP or RFP derivatives in mammalian cells using nanobody binders. Methods 88, 89–97, doi: 10.1016/j.ymeth.2015.06.018 (2015). 26123185

[b38] de Weers M. . Daratumumab, a novel therapeutic human CD38 monoclonal antibody, induces killing of multiple myeloma and other hematological tumors. J Immunol 186, 1840–1848, doi: jimmunol.1003032/jimmunol.1003032 (2011). 21187443 10.4049/jimmunol.1003032

[b39] Deckert J. . SAR650984, a novel humanized CD38-targeting antibody, demonstrates potent antitumor activity in models of multiple myeloma and other CD38+ hematologic malignancies. Clinical cancer research : an official journal of the American Association for Cancer Research 20, 4574–4583, doi: 10.1158/1078-0432.CCR-14-0695 (2014). 24987056

[b40] Goldmacher V. S. . Anti-CD38-blocked ricin: an immunotoxin for the treatment of multiple myeloma. Blood 84, 3017–3025 (1994). 7524764

[b41] Bolognesi A. . CD38 as a target of IB4 mAb carrying saporin-S6: design of an immunotoxin for ex vivo depletion of hematological CD38+ neoplasia. Journal of biological regulators and homeostatic agents 19, 145–152 (2005). 16602630

[b42] Gerdts J., Brace E. J., Sasaki Y., DiAntonio A. & Milbrandt J. SARM1 activation triggers axon degeneration locally via NAD(+) destruction. Science 348, 453–457, doi: 10.1126/science.1258366 (2015). 25908823 PMC4513950

[b43] Tran M. . Production of unique immunotoxin cancer therapeutics in algal chloroplasts. Proceedings of the National Academy of Sciences of the United States of America 110, E15–22, doi: 10.1073/pnas.1214638110 (2013). 23236148 PMC3538218

[b44] Spiess C., Zhai Q. T. & Carter P. J. Alternative molecular formats and therapeutic applications for bispecific antibodies. Molecular Immunology 67, 95–106, doi: 10.1016/j.molimm.2015.01.003 (2015). 25637431

[b45] Cong L. & Zhang F. Genome engineering using CRISPR-Cas9 system. Methods in molecular biology 1239, 197–217, doi: 10.1007/978-1-4939-1862-1_10 (2015). 25408407

[b46] Zhang H. . Dynamic conformations of the CD38-mediated NAD cyclization captured in a single crystal. Journal of molecular biology 405, 1070–1078, doi: 10.1016/j.jmb.2010.11.044 (2011). 21134381 PMC3019291

[b47] Otwinowski Z. & Minor W. Processing of X-ray diffraction data collected in oscillation mode. Method Enzymol 276, 307–326, doi: 10.1016/S0076-6879(97)76066-X (1997). 27754618

[b48] Mccoy A. J. . Phaser crystallographic software. J Appl Crystallogr 40, 658–674, doi: 10.1107/S0021889807021206 (2007). 19461840 PMC2483472

[b49] Bailey S. The Ccp4 Suite - Programs for Protein Crystallography. Acta Crystallogr D 50, 760–763 (1994). 15299374 10.1107/S0907444994003112

[b50] Zhang W. Z., Zhang H. M., Zhang T., Fan H. F. & Hao Q. Protein-complex structure completion using IPCAS (Iterative Protein Crystal structure Automatic Solution). Acta Crystallogr D 71, 1487–1492, doi: 10.1107/S1399004715008597 (2015). 26143920

[b51] Murshudov G. N., Vagin A. A. & Dodson E. J. Refinement of macromolecular structures by the maximum-likelihood method. Acta Crystallogr D 53, 240–255, doi: Doi 10.1107/S0907444996012255 (1997). 15299926

[b52] Emsley P., Lohkamp B., Scott W. G. & Cowtan K. Features and development of Coot. Acta Crystallogr D 66, 486–501, doi: 10.1107/S0907444910007493 (2010). 20383002 PMC2852313

[b53] Painter J. & Merritt E. A. TLSMD web server for the generation of multi-group TLS models. J Appl Crystallogr 39, 109–111, doi: 10.1107/S0021889805038987 (2006).

[b54] Chen V. B. . MolProbity: all-atom structure validation for macromolecular crystallography. Acta Crystallogr D 66, 12–21, doi: 10.1107/S0907444909042073 (2010). 20057044 PMC2803126

[b55] Graeff R. M., Walseth T. F., Fryxell K., Branton W. D. & Lee H. C. Enzymatic synthesis and characterizations of cyclic GDP-ribose. A procedure for distinguishing enzymes with ADP-ribosyl cyclase activity. J Biol Chem 269, 30260–30267 (1994). 7982936

[b56] Graeff R. M., Mehta K. & Lee H. C. GDP-ribosyl cyclase activity as a measure of CD38 induction by retinoic acid in HL-60 cells. Biochemical and biophysical research communications 205, 722–727, doi: 10.1006/bbrc.1994.2725 (1994). 7999103

