# Supplementary material for: Immuno-targeting the multifunctional CD38 using nanobody
Source: Sci Rep. 2016 Jun 2;6:27055. doi: 10.1038/srep27055 (PMC4890012; doi:10.1038/srep27055)

**Immuno-targeting the multifunctional CD38 using nanobody**

Ting **Li**^1^, Shali **Qi**^2^, Mandy **Unger**^3^, Yun Nan **Hou**^1^, Qi Wen **Deng**^1^, Jun **Liu**^1^, Connie MC **Lam**^2^, Xian Wang **Wang**^2^, Xin **Du**^4^, Peng **Zhang**^5^, Friedrich **Koch-Nolte**^3^, Quan **Hao**^2^, Hongmin **Zhang**^6^*, Hon Cheung **Lee**^1^*, Yong Juan **Zhao**^1^*

**Supplementary Information**

**
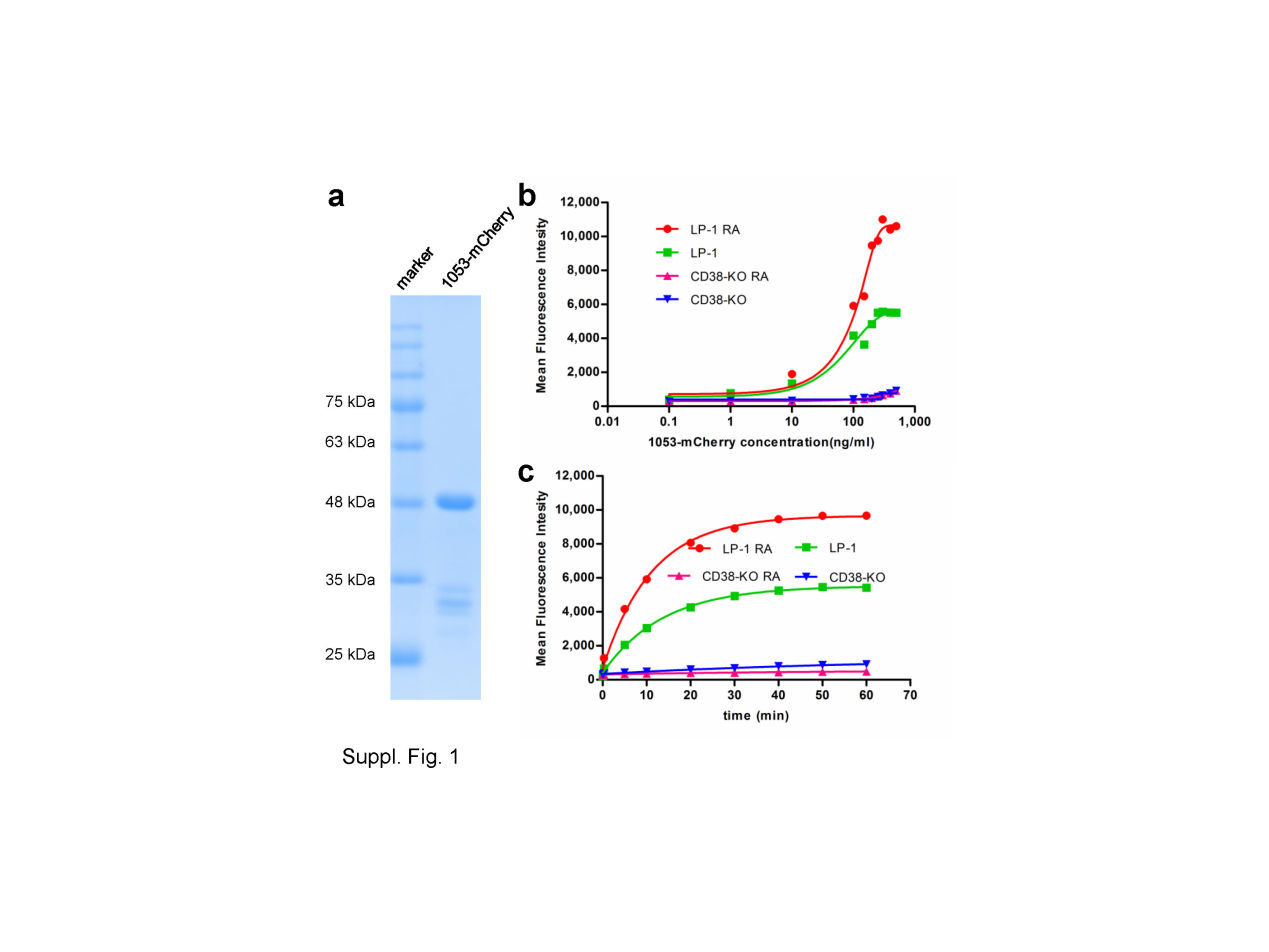
**

**Suppl. Figure 1. Preparation and characterization of the red chromobody, 1053-mCherry.** To test the secretory expression of chromobody in yeast, Nb1053, mCherry and His_6_-tag were subcloned into the yeast expression vector pPICZαA in frame. The resulted plasmid was transformed into Pichia Pastoris. The recombinant proteins were purified from the media after 72 h of induction by methanol. Around 4 mg of 1053-mCherry was obtained from 2 L culture after one step Ni-NTA column purification. (a) SDS-PAGE analysis of the pure 1053-mCherry. Dose curve (b) and time course (c) of live cell staining analyzed by FACS. A series of concentrations of 1053-mCherry stained LP-1 and CD38-KO cell line, with or without RA pre-treatment, at 4℃ for 30 min (red: LP-1 RA; green: LP-1; pink: CD38-KO RA; blue: CD38-KO). RA pre-treatment: 5x10^5^ cells/ml, 10 nM RA for 3 days; initial cell density for the staining was 5x10^5^ cells/ml. The experiments were repeated four times.


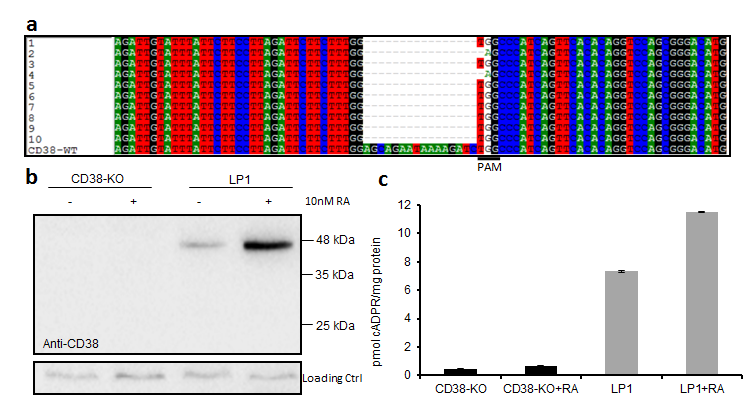
**Suppl. Figure 2. Construction and validation of CRISPR/Cas9 plasmids for CD38 in LP-1 cells.** The guide RNA (gRNA, TTGGAGCAGAATAAAAGATCTGG) targets the region of exon 3 in CD38 gene, which was cloned into the plasmid, lentiCRISPRv2 (52961, Addgene). The gRNA is under the control of a U6 promoter and a CMV promoter drives the expression of the Cas9 enzyme. To produce lentiviral particles, HEK293T cells were grown to 70%-90% confluence and transfected with the CRISPR plasmid, psPAX2 packaging and pMD2.G envelope plasmids using Lipofectamine^TM^ 2000 Reagent (Life Technology) according to the manufacturer’s instruction. Media containing lentiviral particles was harvested 48-72 hours after transfection and followed by infection of LP-1 cells. Selection of positive cells was done in the media containing 1μg/ml puromycin. To obtain the cell line with homozygous deletion of CD38 (CD38-KO), single colonies were selected by serial dilution and validated by DNA sequencing following PCR amplification of genomic DNA using the primer pair (5’-ACTCTCCTGCACACAGAAATCA-3’ and 5’-CATCAGGATGGGTGCCTGTG-3’).

(a) Sequencing data of the CD38-KO cell line shows that there are two different kinds of deletion, with the length of 16-bp and 17-bp, probably in two different chromosomes. (b) Expression level of CD38 in both wildtype and CD38-KO LP-1 cells, with or without RA-treatment (10nM, 3 days) blotted by anti-CD38. The result shows that no CD38 was detectable in the knockout cells. The nonspecific band serves as the loading control. (c) Intracellular cADPR level measured by the cycling assay[^1^](#_ENREF_1) was much lower in CD38-KO cells than that in wildtype cells with or without RA treatment, indicating the deletion of CD38, the main synthesizing enzyme of cADPR.

1 Graeff, R. & Lee, H. C. A novel cycling assay for cellular cADP-ribose with nanomolar sensitivity*. The Biochemical journ*a**l 3**61, 379-384 (2002).

**
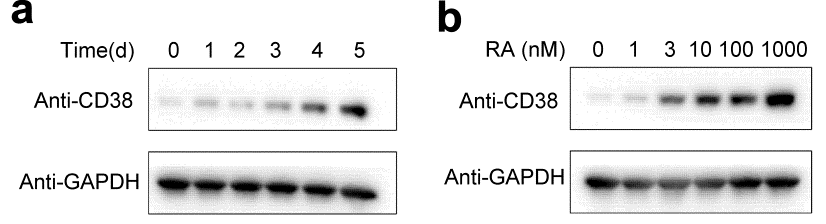
**

**Suppl. Figure 3. CD38 expression induced by RA.** (a) Time course of CD38 expression in LP-1 cells induced with 10 nM RA for 0, 1, 2, 3, 4, 5 days and blotted by anti-CD38. Anti-GAPDH blots were used as internal controls. (b) Dose-dependence analysis of CD38 expression in LP-1 cells induced with different concentrations of RA for 3 days. The experiments were repeated three times.

**
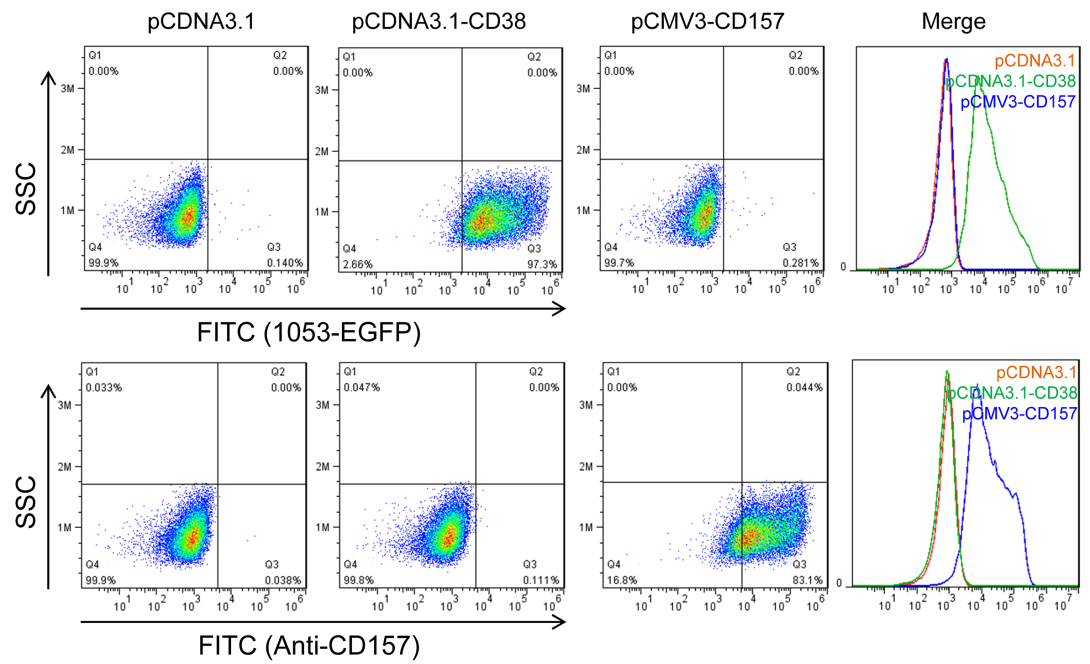
**

**Suppl. Figure 4. Cross-reactivity of CD38 chromobody against CD157.** HEK 293T cells were transfected with pcDNA3.1 (control, empty vector), pcDNA3.1-CD38 (vector containing CD38) or pCMV3-CD157 (vector containing CD157, Sino Biological Inc, China), respectively. Forty hours post-transfection, the cells were stained with 1053-EGFP (0.5 μg/ml) or anti-CD157 (1 μg/ml, gifted from Prof. Fabio Malavasi of University of Torino Medical School). The staining of anti-CD157 was visualized and amplified by anti-Mouse-FITC. Flow cytometry and analysis were done as described in Methods. The experiments were repeated three times.

The data show that 1053-EGFP does not recognize CD157 (upper panel, 3^rd^ and 4^th^ charts), which expression was confirmed by anti-CD157 staining (lower panel, 3^rd^ and 4^th^ charts). HEK 293T cells express neither CD38 nor CD157. The results indicate no cross-reactivity was found between CD38/CD157 antibodies and CD157/CD38 antigens.

**
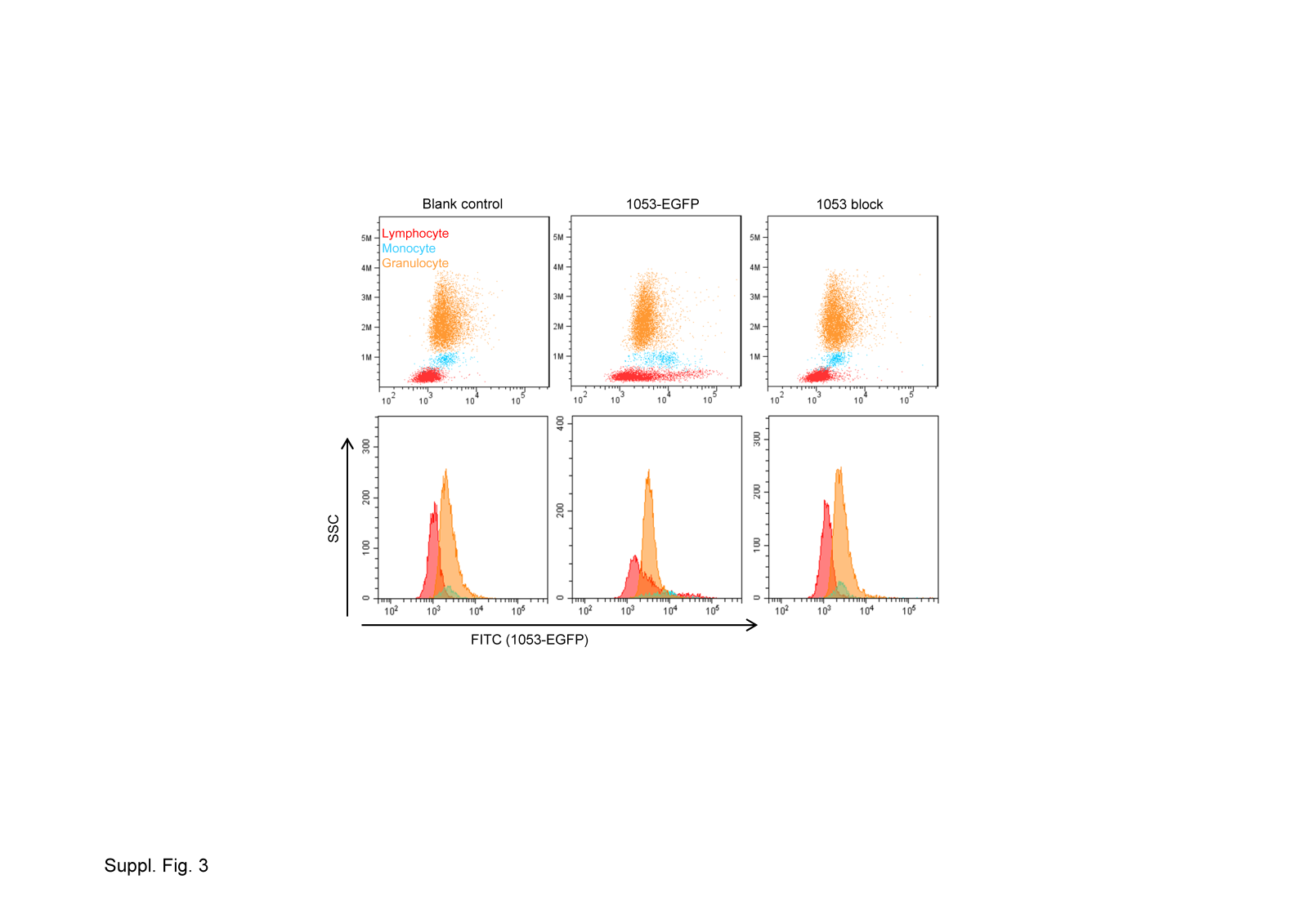
**

**Suppl. Figure 5. CD38 expression on the surface of normal PWBCs.** PWBCs were stained by 1053-EGFP and analyzed by FACS. Three different populations in PWBCs (Lymphocytes: red; monocytes: cyan; granulocytes: orange) were gated according to FSC and SSC. The mean of green fluorescence intensity of the different populations was plotted against SSC (upper) or in histogram (lower). Blank control: incubated with vehicle; 1053-EGFP: incubated with 500 ng/ml 1053-EGFP at 4℃ for 30 min; 1053 block: pre-incubated with 2 μg/ml of 1053 (free nanobody, serves as a competitor of 1053-EGFP) at 4℃ for 30 min followed by 1053-EGFP staining. The experiments were repeated three times. The results indicate that both monocytes and lymphocytes express more CD38 than granulocytes.

**
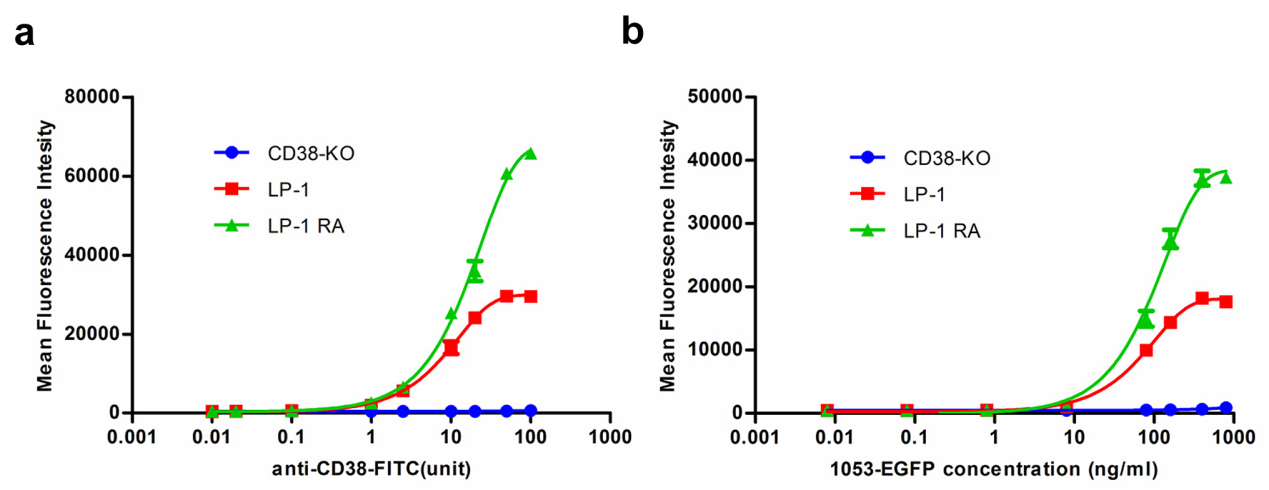
**

**Suppl. Figure 6. Staining cell surface CD38 by the commercial anti-CD38-FITC and 1053-EGFP.** Dose curves of staining wildtype and CD38-KO LP-1 cells with or without RA-treatment by anti-CD38-FITC (a) and 1053-EGFP (b). The method is similar to Fig. 3d. In chart (a), 100 units equal to the amount of 1 test (10 μl), defined by the manufacture (Beckman Coulter). And the volume and cell number were similar to those for chromobody staining. n=3.

**
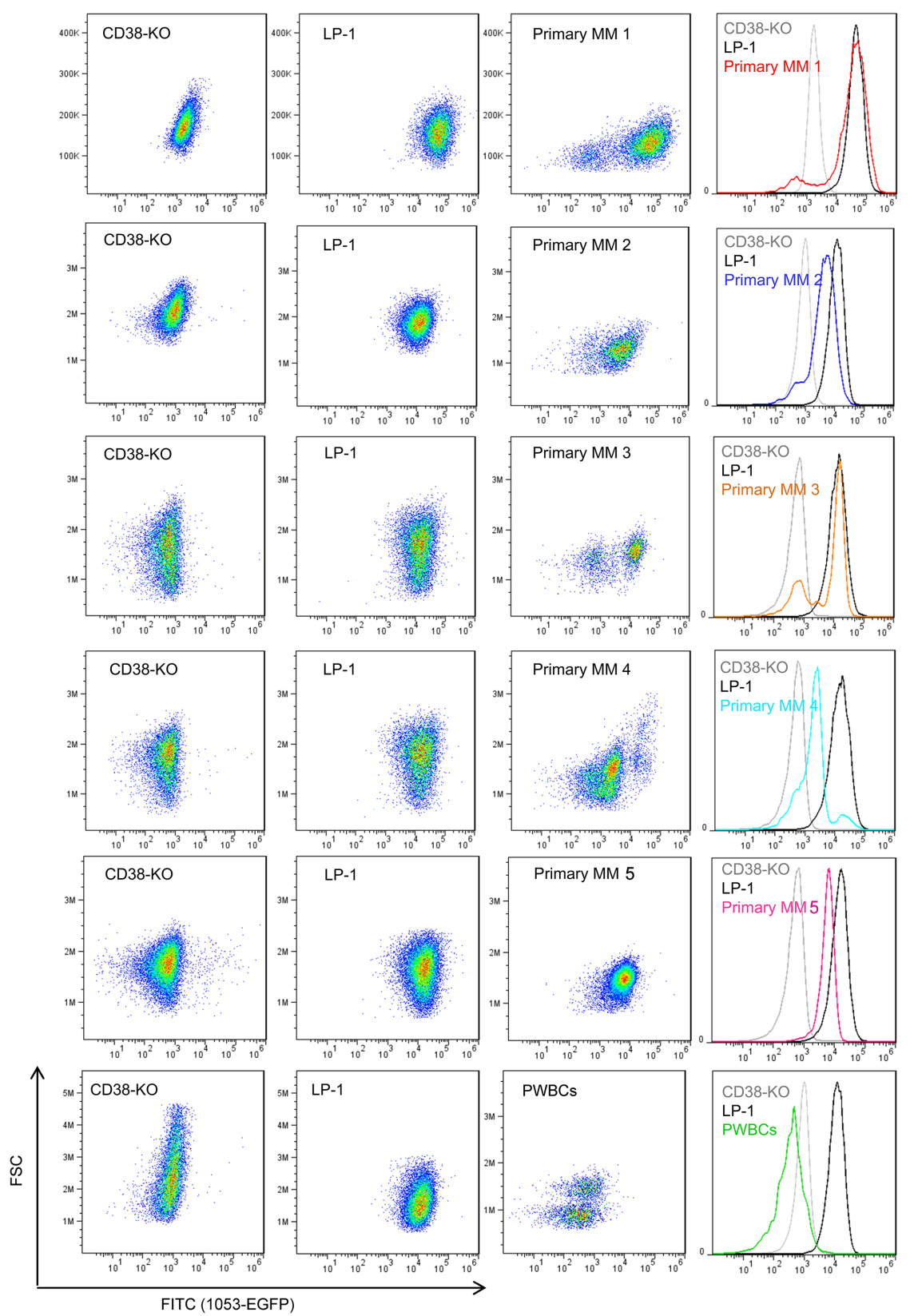
**

**Suppl. Figure 7. Dot plot data of Fig. 6a.**

**
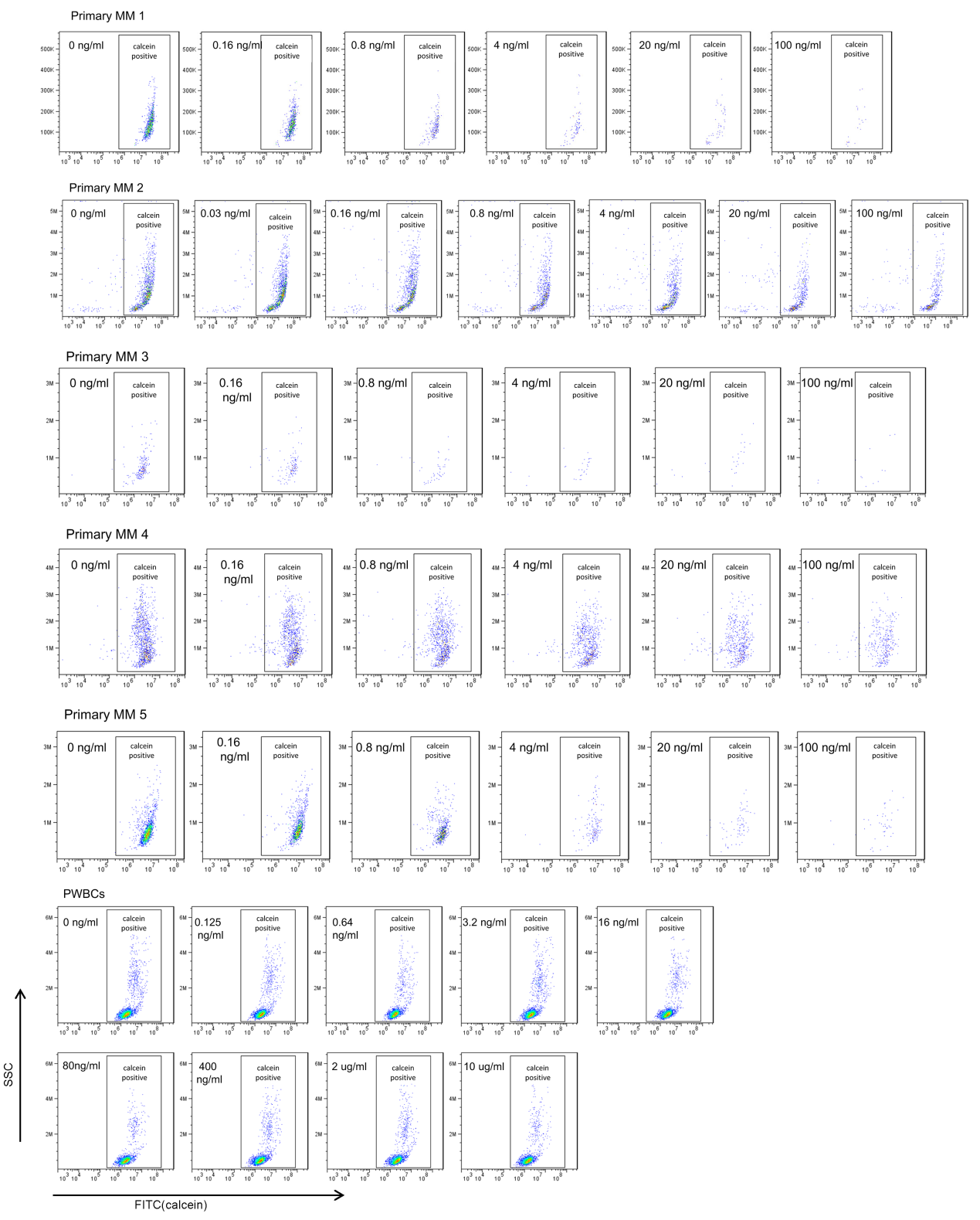
**

**Suppl. Figure 8. Dot plot data of Fig. 6b.** The numbers shown in the upper right corners are the corresponding concentrations of 1053-PE38.


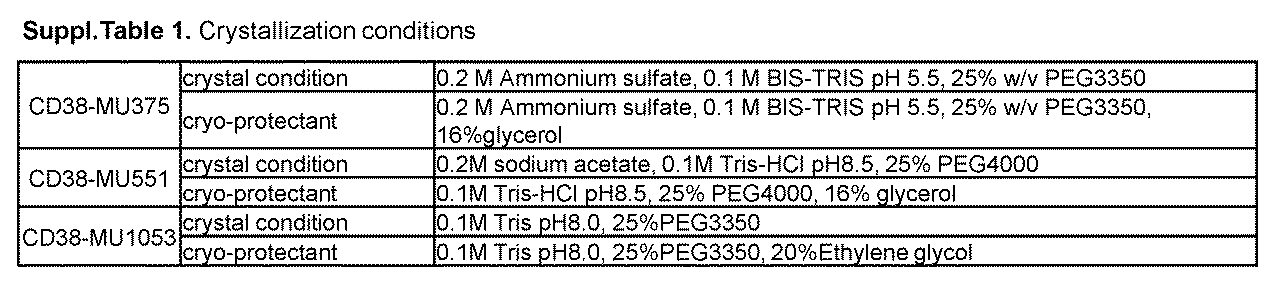

Supplement: Supplementary Information [file srep27055-s1.doc]
